# Monitoring data of the openLAB research bridge – Part 1: Reference condition

**DOI:** 10.1016/j.dib.2025.111624

**Published:** 2025-05-07

**Authors:** Andreas Jansen, Max Herbers, Bertram Richter, Maria Walker, Frank Jesse, Steffen Marx

**Affiliations:** a MKP GmbH, Zum Hospitalgraben 2, Weimar 99425, Germany; b Institute of Concrete Structures, TUD Dresden University of Technology, Dresden 01062, Germany; c Hentschke Bau GmbH, Zeppelinstraße 15, Bautzen 02625, Germany

**Keywords:** Civil engineering, Structural health monitoring, Sensor faults, Fault diagnosis, Damages

## Abstract

Structural Health Monitoring (SHM) is emerging as an essential tool for ensuring the safety and longevity of an aging bridge infrastructure. Recent collapses, such as the Morandi Bridge in Genoa, Italy (2018), and the Carola Bridge in Dresden, Germany (2024), emphasize the urgent need for reliable methods to detect early signs of structural deterioration to prevent catastrophic failures. However, the availability of real-world data for developing and particularly validating these methods remains limited. To address this gap, a 45-m-long prestressed concrete (PC) bridge – the openLAB bridge – has been built as part of the IDA-KI research project, designed to simulate common structural deficiencies as well as sensor faults. The bridge is equipped with a comprehensive SHM system, including fiber optic and electrical sensors, to continuously monitor its behavior. This dataset provides researchers with a unique opportunity to improve damage detection models, validate SHM methods, and ultimately enhance infrastructure safety. This first publication includes data from the undamaged bridge, covering the first nine months from February 1, 2024, to October 31, 2024, under monthly simulated traffic loads. It features measurements from the electrical monitoring system, including acceleration, tilt, air temperature, humidity, and solar radiation. After a one-year reference phase, the bridge will undergo controlled damaging load tests. The dataset will be periodically updated, providing insights into both undamaged and damaged states.

Specifications Table

**Table:** 

Subject	Civil and Structural Engineering
Specific subject area	Structural Health Monitoring of Bridges
Type of data	Sensor data as Comma Separated Value (CSV) text files
Data collection	The presented data is collected using a Gantner Instruments system (Q.station 101T, Q.bloxx modules) with the following sensors: 6 IEPE-accelerometers (PCB 393A03), 6 digital MEMS-tiltmeters with integrated temperature and humidity sensors (Sisgeo 0S542HD0502), 1 temperature and humidity sensor (Thies Clima 1.1005.54.773), and 1 pyranometer (Kipp & Zonen SP Lite2). All sensors, except accelerometers, sample every 10 min. Additionally, event-triggered measurements are collected for accelerometers and tiltmeters
Data source location	Collection: Hentschke Bau GmbH, Hoyerswerdaer Str. 42, 02625 Bautzen Storage: MKP GmbH, Zum Hospitalgraben 2, 99425 Weimar, Germany
Data accessibility	Repository name: Open Access Repository and Archive (OPARA) Data identification number: 10.25532/OPARA-660 Direct URL to data: https://opara.zih.tu-dresden.de/handle/123456789/1048 The data is licenced under CC-BY-SA 4.0
Related research article	Herbers, M.; Richter, B.; Bartels, J.; Al-Zuriqat, T.; Smarsly, K.; Marx, S.: openLAB – A large-scale demonstrator for advancing digital twin developments of bridges. In: Henry, R.S.; Palermo, A. (Ed.): ReConStruct: Resilient Concrete Structures, Proc. of the 2024 fib Symposium. 11 November 2024 in Christchurch (New Zealand), 2024, S. 2057-2067 – 978-2-940643-25-7

## 1 Value of the Data

**Fig. 1:**
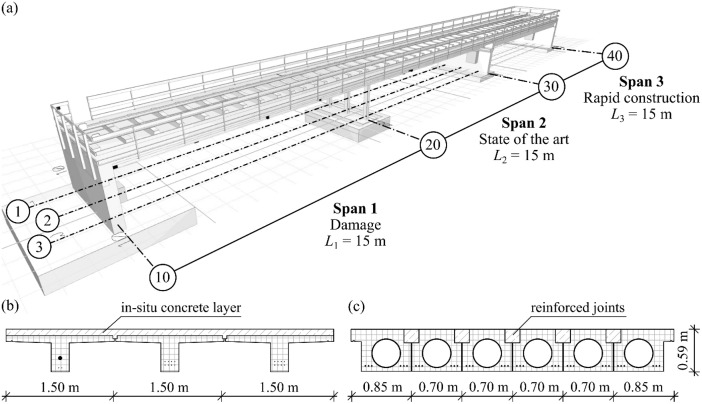
Illustration of the openLAB bridge: (a) overview (b) cross-section of span 1 (c) cross-section of span 3 (Figure: Fabian Collin, Max Herbers).
• To establish a robust baseline for data-driven SHM methods based on probability and machine learning, e.g. [5,6], damaging load tests will begin only after a one-year reference period. This phase is essential for characterizing the bridge’s undamaged condition and capturing its behavior under varying environmental conditions [7]. Subsequent structural damage can be identified by observing deviations from this baseline condition. Additionally, the dataset includes data from sensor malfunctions, providing a valuable opportunity to develop data-driven approaches that can reliably distinguish between anomalies caused by structural damage and those resulting from sensor faults.
• While data-driven SHM can identify anomalies that may indicate structural damage, physics-based approaches, particularly those utilizing Finite Element (FE) models, are best suited to assess the extent and consequences of such damage [8,9]. Recent research has also incorporated environmental variability, such as temperature effects, into these models [10]. The published dataset enhances research on physics-based SHM by providing monitoring data for FE model calibration throughout the experiment. The dataset includes key construction plans for developing an FE model, with further details available upon request.
• Monthly controlled load tests are conducted to support the development of SHM methods based on traffic loads. These tests provide essential data to study the relationship between traffic loading and structural response. Recent research demonstrates promising developments in data-driven approaches that utilize features derived from unknown traffic loads, such as the R-Signature [11,12] and the niil-feature [13]. Simulated data in [12] has shown that the R-Signature is significantly more sensitive to damage compared to natural frequencies. The data from the openLAB bridge [14] offers a valuable opportunity to validate these findings on a real structure.
• Ultimately, the comprehensive design of the monitoring system and the damage scenarios allow different SHM methods to be benchmarked against each other. Researchers can use the dataset to compare the reliability, robustness, effort, and costs of their methods in identifying certain types of structural defects. This comparison facilitates a deeper understanding of the strengths and limitations of each approach, enabling the optimization and selection of the most suitable methods for practical SHM applications.

• Transportation infrastructure forms the backbone of modern societies but faces increasing strain from rising traffic loads, progressive aging, and insufficient maintenance investment. Efficient Structural Health Monitoring (SHM) can be a key tool to detect anomalies at an early stage and to initiate targeted maintenance measures. However, reliably distinguishing between sensor faults and actual structural damage remains challenging. This is partly due to the lack of validation data from damaged structures under real-world conditions. Such validation is problematic because intentionally damaging an in-service bridge for research purposes is infeasible. Consequently, very few datasets exist that coincidentally capture damage events during monitoring campaigns, e.g., [1], and even fewer are publicly available. Some research projects had the opportunity to damage a large-scale bridge at the end of its service life, e.g., [2,3]. The resulting data is often limited to a specific sensor type, mostly accelerometers, and only a few damage scenarios. The openLAB dataset presented here is a valuable contribution to the research community as it incorporates diverse sensor types and investigates multiple damage scenarios, providing a comprehensive resource for validating SHM methods.
• The openLAB bridge, introduced in [4], is a prestressed concrete (PC) structure constructed using precast elements (PEs), reflecting the widespread use of PC bridges in the infrastructure networks of many countries. The bridge consists of three spans, each measuring 15 m, with distinct research focuses for each span, see Fig. 1. These design choices allow for a diverse range of damage scenarios. The following damage tests are planned: (i) Selected girders will be loaded up to their Ultimate Limit State (ULS), (ii) individual tendons will be intentionally damaged, and (iii) static and dynamic tests will be conducted in the area of the coupling joint and in critical shear zones.

## 2 Background

Relevant background is provided in the previous section.

## 3 Data Description

The openLAB-bridge shown in Fig. 1 is equipped with various monitoring systems combining fiber optic and electrical sensors to capture both structural behavior and environmental conditions. This initial dataset [14] contains only data acquired from an electrical measurement system from Gantner Instruments (Q.station 101T, with various Q.bloxx modules).

The dataset contains measurements of acceleration, tilt, air temperature, humidity, and solar radiation. Data is continuously recorded at 10-min intervals, with additional triggered measurements during non-damaging load tests conducted using a test vehicle or in response to elevated vibration levels caused by road traffic or nearby construction activities. The published repository provides the data in Comma Separated Value (CSV) format. Each file includes a header specifying the names of the data columns. Additional details, such as units and sampling frequency, are provided in the accompanying README file or this document. Each CSV file contains a *Timestamp* column that records the time of each sample as a datetime string in ISO 8601 format, without time zone information. All timestamps are in Coordinated Universal Time (UTC). Sensor data is represented as decimal numbers.

### 3.1 Data Column Naming Convention

**Fig. 2:**
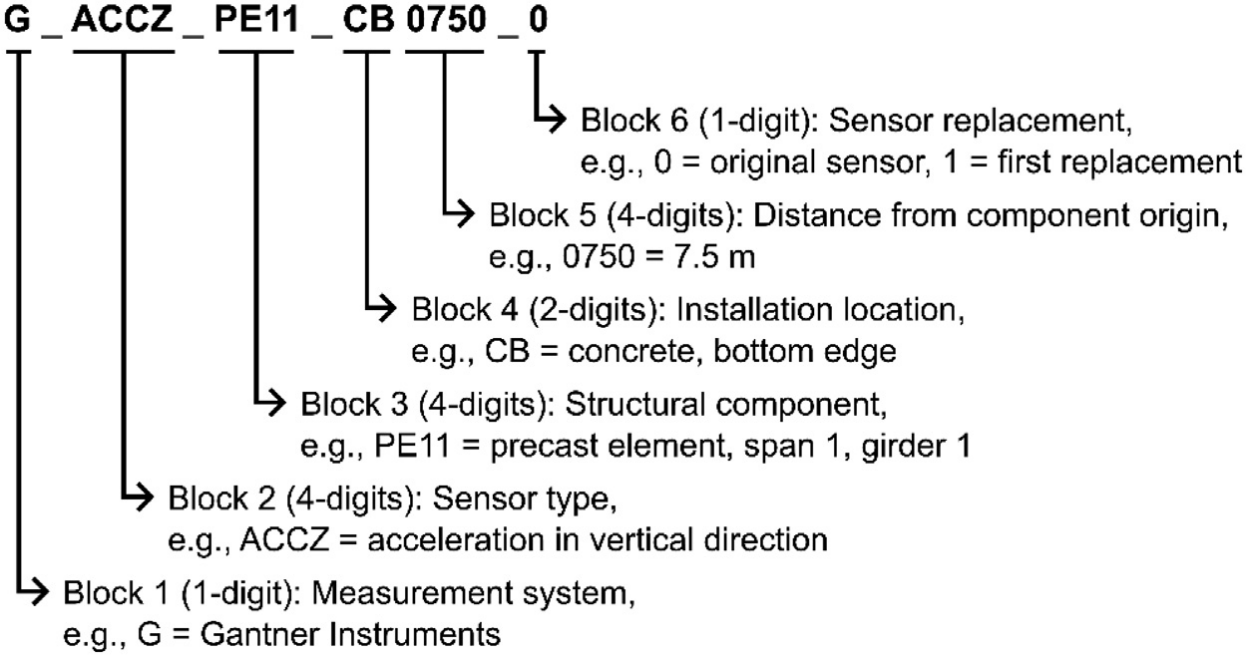
Illustration of data column naming convention for dataset.

To ensure consistency across the various measurement systems and sensor types, a standardized naming convention has been established for the resulting data columns. Each 20-character column name encodes key information, including the measurement system, measurement type, structural component, and sensor installation location. Certain sensor products offer multiple measurement capabilities, such as simultaneously recording tilt and temperature. Consequently, data from these sensors is represented in multiple data columns. The naming convention is illustrated in Fig. 2 using the example of the data column *G_ACCZ_PE11_CB_0750_0*.

The options for each block in the naming scheme are listed in Table 1. Only the relevant options for the presented dataset are included. For example, since the dataset does not contain data from Distributed Fiber Optic Sensors (DFOS) and exclusively uses the electrical Gantner Instruments measurement system, the first block of each column name is always represented by *G*. This block has been omitted from the table for brevity.

**Table 1:** Options for each block in data column naming convention.

Block	Abbreviation	Description
2 - Measurement type	ACCZ	Acceleration in vertical direction
	HTST	Hygro-thermo sensor/temperature
	HTSH	Hygro-thermo sensor/humidity
	PYRS	Pyranometer
	TILY	Tiltmeter/tilt in longitudinal bridge direction
	TILX	Tiltmeter/tilt in transverse bridge direction
	TILT	Tiltmeter/temperature
	TILH	Tiltmeter/humidity

3 - Structural component (refer to Fig. 1)	PE11	Precast element, span 1 girder 1
	PE12	Precast element, span 1 girder 2
	PE13	Precast element, span 1 girder 3
	PE21	Precast element, span 2 girder 1
	PE22	Precast element, span 2 girder 2
	PE23	Precast element, span 2 girder 3
	ENVR	Environment

4 - Installation location	CB	Concrete, bottom edge
	EN	Environment

The repository comprises four main data directories and one supplementary material directory. The contents are described in detail in the following sections.

### 3.2 Directory: *01_acceleration_trigger*

**Fig. 3:**
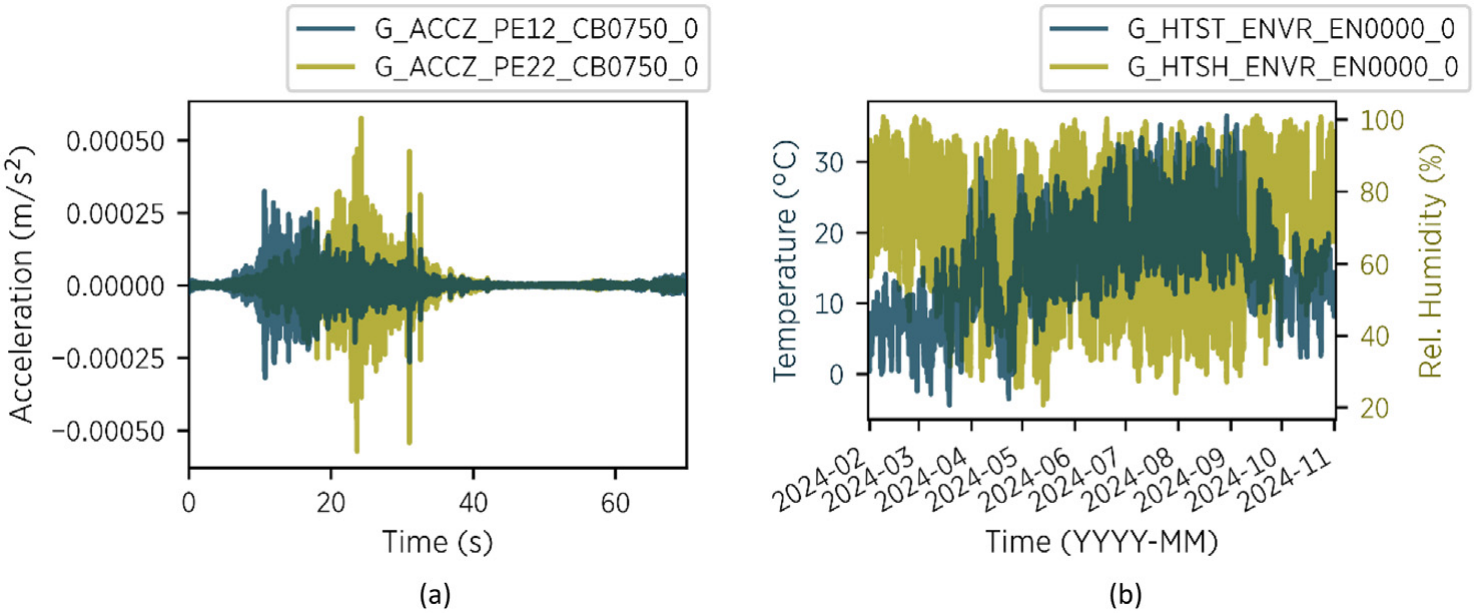
Examples of measurement data: (a) signals of two sensors from a 70-second sample of the triggered acceleration measurement – file: 01_acceleration_trigger/acc_2024_09_18_T10_15_11.csv (b) temperature and humidity from 2024-02-01 to 2024-10-31 – files: 02_environment/ environment_2024_02.csv to environment/ environment_2024_10.csv.

The directory *01_acceleration_trigger* contains triggered acceleration measurements. As the openLAB bridge shows very low acceleration response to ambient excitation, a 70-s sample is collected whenever the bridge exhibits increased vibration activity. Specifically, a measurement is triggered when the signal from sensor *G_ACCZ_PE11_CB0750_0* exceeds 2 × 10⁻⁴ m/s² within the considered time period. The trigger system was activated on 2024-05-01, and data is available for the period from 2024-05-01 to 2024-10-31. An example of the acceleration data of two sensors can be found in Fig. 3(a).

**Files:** The directory contains 523 CSV files. Each file is named using the prefix *acc* followed by the timestamp marking the start of the triggered measurement. The file naming convention is as follows: *acc_{year}_{month}_{day}_T{hour}_{minute}_{second}.csv*

**Sensors:** PCB 393A03

**Sample rate:** 500 Hz

**Schema:** The schema of the CSV files is described in Table 2.

**Table 2:** Schema of CSV files in the directory *01_acceleration_trigger.*

Column name	Data type	Description
Timestamp	Datetime	ISO 8601 format (UTC)
G_ACCZ_PE11_CB0750_0	Decimal Number	Acceleration in m/s²
G_ACCZ_PE12_CB0750_0	Decimal Number	Acceleration in m/s²
G_ACCZ_PE13_CB0750_0	Decimal Number	Acceleration in m/s²
G_ACCZ_PE21_CB0750_0	Decimal Number	Acceleration in m/s²
G_ACCZ_PE22_CB0750_0	Decimal Number	Acceleration in m/s²
G_ACCZ_PE23_CB0750_0	Decimal Number	Acceleration in m/s²

**Preprocessing:** The median of each measurement has been subtracted from the signals to correct for non-zero offsets from the piezoelectric sensors. Additionally, a fourth-order Butterworth filter with a low-frequency cutoff of 0.5 Hz and a high-frequency cutoff of 100 Hz has been applied. The low-frequency cutoff was selected based on the measurement range specified by the sensor manufacturer, while the high-frequency cutoff serves as an anti-aliasing measure.

### 3.3 Directory: *02_environment*

The *02_environment* directory contains measurements from air temperature, humidity, and solar radiation sensors. These sensors are part of a climate station mounted on top of the bridge. Samples are collected in 10-min intervals, with data available from 2024-02-01 to 2024-10-31. Temperature and humidity data are illustrated in Fig. 3(b).

**Files:** The directory contains 9 CSV files each containing the data for one month. The file naming convention is as follows: *environment_{year}_{month}.csv*

**Sensors:** Thies Clima 1.1005.54.773, Kipp & Zonen SP Lite2

**Sample rate:** 1/600 Hz

**Schema:** The schema of the CSV files is described in Table 3.

**Table 3:** Schema of CSV files in the directory *02_environment.*

Column name	Data type	Description
Timestamp	Datetime	ISO 8601 format (UTC)
G_HTST_ENVR_EN0000_0	Decimal Number	Air temperature in°C
G_HTSH_ENVR_EN0000_0	Decimal Number	Relative humidity in %
G_PYRS_ENVR_EN0000_0	Decimal Number	Solar radiation in W/m²

**Preprocessing:** No preprocessing steps have been applied.

### 3.4 Directory: *03_tiltmeter*

**Fig. 4:**
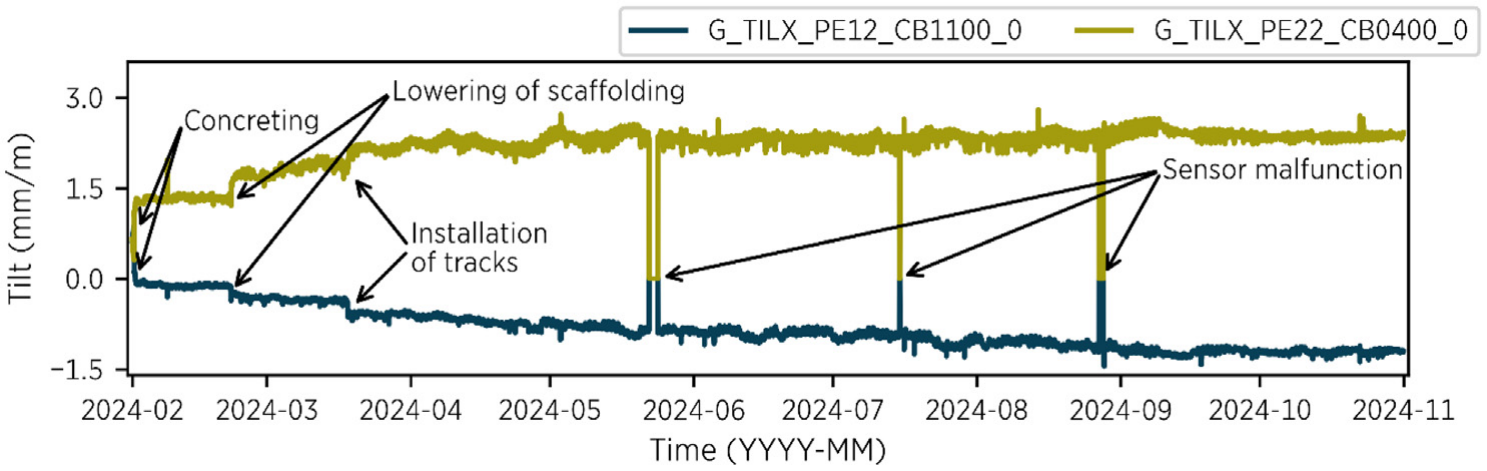
Measurement data for two tiltmeters from 2024-02-01 to 2024-10-31 – files: 03_tiltmeter/tiltmeter_2024_02.csv to tiltmeter/tiltmeter_2024_10.csv. Significant events are marked.

Tiltmeter data is recorded every 10 min and can be found in the *03_tiltmeter* directory. Tilt is measured along the transverse bridge direction (TILY) and the longitudinal bridge direction (TILX). Additionally, each tiltmeter records air temperature and relative humidity. Data is available from 2024-02-01 to 2024-10-31. Fig. 4 illustrates the records of two tiltmeters, highlighting significant events such as the lowering of the scaffolding. For a comprehensive documentation of the construction process, refer to Table 6.

**Files:** The directory contains 9 CSV files each containing the data for one month. The file naming convention is as follows: *tiltmeter_{year}_{month}.csv*

**Sensors:** Sisgeo 0S542HD0502

**Sample rate:** 1/600 Hz

**Schema:** The schema of the CSV files is described in Table 4.

**Table 4:** Schema of CSV files in the directory *03_tiltmeter.*

Column name	Data type	Description
Timestamp	Datetime	ISO 8601 format (UTC)
G_TILY_PE11_CB1100_0	Decimal Number	Tilt in mm/m along the transverse direction
G_TILX_PE11_CB1100_0	Decimal Number	Tilt in mm/m along the longitudinal direction
G_TILT_PE11_CB1100_0	Decimal Number	Air temperature in°C
G_TILH_PE11_CB1100_0	Decimal Number	Relative humidity in %
G_TILY_PE12_CB1100_0	Decimal Number	Tilt in mm/m along the transverse direction
G_TILX_PE12_CB1100_0	Decimal Number	Tilt in mm/m along the longitudinal direction
G_TILT_PE12_CB1100_0	Decimal Number	Air temperature in°C
G_TILH_PE12_CB1100_0	Decimal Number	Relative humidity in %
G_TILY_PE13_CB1100_0	Decimal Number	Tilt in mm/m along the transverse direction
G_TILX_PE13_CB1100_0	Decimal Number	Tilt in mm/m along the longitudinal direction
G_TILT_PE13_CB1100_0	Decimal Number	Air temperature in°C
G_TILH_PE13_CB1100_0	Decimal Number	Relative humidity in %
G_TILY_PE21_CB0400_0	Decimal Number	Tilt in mm/m along the transverse direction
G_TILX_PE21_CB0400_0	Decimal Number	Tilt in mm/m along the longitudinal direction
G_TILT_PE21_CB0400_0	Decimal Number	Air temperature in°C
G_TILH_PE21_CB0400_0	Decimal Number	Relative humidity in %
G_TILY_PE22_CB0400_0	Decimal Number	Tilt in mm/m along the transverse direction
G_TILX_PE22_CB0400_0	Decimal Number	Tilt in mm/m along the longitudinal direction
G_TILT_PE22_CB0400_0	Decimal Number	Air temperature in°C
G_TILH_PE22_CB0400_0	Decimal Number	Relative humidity in %
G_TILY_PE23_CB0400_0	Decimal Number	Tilt in mm/m along the transverse direction
G_TILX_PE23_CB0400_0	Decimal Number	Tilt in mm/m along the longitudinal direction
G_TILT_PE23_CB0400_0	Decimal Number	Air temperature in°C
G_TILH_PE23_CB0400_0	Decimal Number	Relative humidity in %

**Preprocessing:** No preprocessing steps have been applied.

### 3.5 Directory: *04_tiltmeter_trigger*

In addition to continuous tilt measurements, a 90-s sample is recorded each time a load test is conducted with the test vehicle. Each test follows the same procedure: the vehicle begins in bridge span 3 near the abutment, drives to span 1, stops near the abutment, and then returns to its starting position. Throughout the test, the vehicle moves at a maximum speed of approximately 4 km/h and remains on the bridge for the entire time. Each recording captures a complete test cycle, including crossings in both directions. A threshold trigger is used to start the measurement. The tests are scheduled monthly. However, additional tests may be conducted occasionally, such as during measurement campaigns organized by external research teams.

**Fig. 5:**
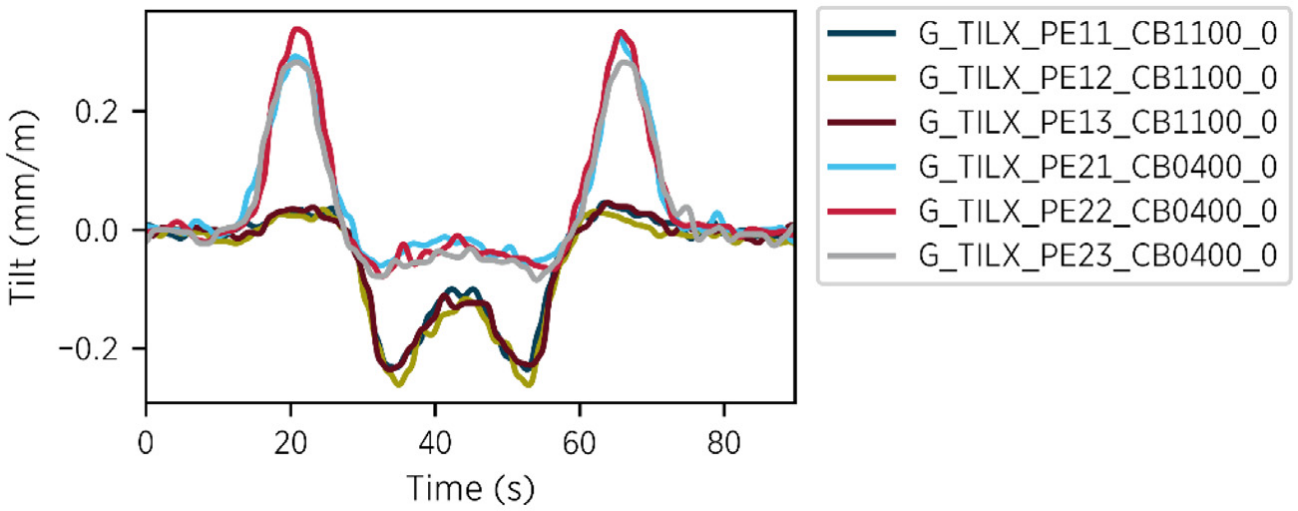
Tilt measurement during a load test with the test vehicle – file: 04_tiltmeter_trigger/tiltmeter_2024_08_28_T10_51_05.csv.

The first load test was conducted on 2024-06-05 and data from all load tests up to 2024-10-31 are included. Only the channels measuring tilt along the bridge's longitudinal direction are recorded during the tests. An example of the tiltmeter data captured during a load test is shown in Fig. 5.

**Files:** The directory contains 151 CSV files. Each file name includes the prefix *tiltmeter* followed by the timestamp marking the start of the triggered measurement. The file naming convention is as follows: *tiltmeter_{year}_{month}_{day}_T{hour}_{minute}_{second}.csv*

**Sensors:** Sisgeo 0S542HD0502

**Sample rate:** 5 Hz

**Schema:** The schema of the CSV files is described in Table 5.

**Table 5:** Schema of CSV files in the directory *04_tiltmeter_trigger.*

Column name	Data type	Description
Timestamp	Datetime	ISO 8601 format (UTC)
G_TILX_PE11_CB1100_0	Decimal Number	Tilt in mm/m along the longitudinal direction
G_TILX_PE12_CB1100_0	Decimal Number	Tilt in mm/m along the longitudinal direction
G_TILX_PE13_CB1100_0	Decimal Number	Tilt in mm/m along the longitudinal direction
G_TILX_PE21_CB0400_0	Decimal Number	Tilt in mm/m along the longitudinal direction
G_TILX_PE22_CB0400_0	Decimal Number	Tilt in mm/m along the longitudinal direction
G_TILX_PE23_CB0400_0	Decimal Number	Tilt in mm/m along the longitudinal direction

**Preprocessing:** To isolate signal components related specifically to the load test, the median of the initial 4 s is subtracted from each channel, removing unrelated influences such as temperature effects. Additionally, to filter out falsely triggered measurements, each sample is compared to a reference crossing using cross-correlation to calculate a time-dependent Pearson coefficient. Only samples with a maximum coefficient above 0.85 are considered valid.

### 3.6 Directory: *05_supplementary_material*

The dataset is accompanied by supplementary materials that provide essential context regarding the bridge structure, the measurement system, and the load testing campaign. This information can be found in the *05_supplementary_material* directory. The content is structured into the following five subdirectories:

• ***01_plans*:** Contains the construction drawings of the openLAB bridge in PDF format. All annotations in the plans are in German.
• ***02_bim*:** Includes detailed 3D models of the openLAB bridge in Industry Foundation Classes (IFC) format - an open, widely supported standard for Building Information Modeling (BIM). These files can be viewed with any standard IFC viewer (many are freely available). Sub-models are included for the bridge geometry, the monitoring system, the reinforcement layout, the scaffolding, and the test vehicle. Together, these models supply the technical detail needed to support advanced numerical simulations, such as reproducing sensor signals from the load testing campaigns.
• ***03_sensor_datasheets*:** Contains the technical datasheets of all sensors used in the monitoring system, provided as PDF files.
• ***04_sensor_installation*:** Includes photographs in JPG format documenting the sensor installation process, with brief descriptions available in the accompanying *captions.md* file.

***05_website*:** A broader overview of the openLAB bridge research project is available on the official project website [15]. To ensure long-term accessibility, an offline copy is included in this directory. The copy is provided in HTML format and can be viewed in any web browser. The website also contains a short video tutorial (with English subtitles) explaining how to navigate and use the IFC models.

## 4 Experimental Design, Materials and Methods

In the following, additional details about the openLAB bridge, its construction process, and the installed monitoring system are provided. This information forms the basis for understanding and interpreting the presented dataset.

### 4.1 openLAB – A Research Bridge

The openLAB bridge serves as a large-scale real-world laboratory where researchers can evaluate SHM methods and non-destructive testing techniques. Given the high prevalence of PC bridges in infrastructure networks, the research bridge was constructed using PC. Located in Bautzen, Germany, the bridge comprises three spans, each measuring 15 m in length. Various common construction techniques are employed, with each span addressing distinct research objectives, see Fig. 1:

• Span 1 includes typical structural deficiencies observed in early PC constructions, such as coupling joint issues, stress corrosion cracking, and regions with reduced shear capacity.
• Span 2 represents modern construction techniques using PEs. After assembling the PEs on site, an additional in-situ concrete layer was poured on both spans 1 and 2 to achieve continuous load-bearing behavior in both longitudinal and transverse directions.
• Span 3 is currently decoupled and employs a rapid construction system using PC girders. To reduce the CO₂ footprint, cylindrical hollow steel bodies were integrated into the PEs as permanent formwork. Transverse load distribution is ensured by cast-in-place reinforced joints, allowing the structure to be fully loadable almost immediately after installation.

Additionally, various intentional defects, such as gravel pockets and cavities, were incorporated into all spans to simulate real-world conditions.

By default, all PEs were pretensioned. However, a combination of pretensioning and posttensioning was applied for PE 1.1 and 2.1. PE 1.1 was designed to simulate coupling joint problems and was therefore fabricated in two separate sections. In PE 2.1, a so-called *smart tendon* was installed, integrating DFOS into the prestressing strands to monitor the strain distribution and its evolution over time [16].

More detailed information on the bridge can be found in [4]. Technical drawings, BIM models, and additional documentation are available in the dataset as well as via the EPLASS InfoClient. Instructions for accessing the InfoClient can be found on the openLAB website [15].

### 4.2 Construction Process

**Fig. 6:**
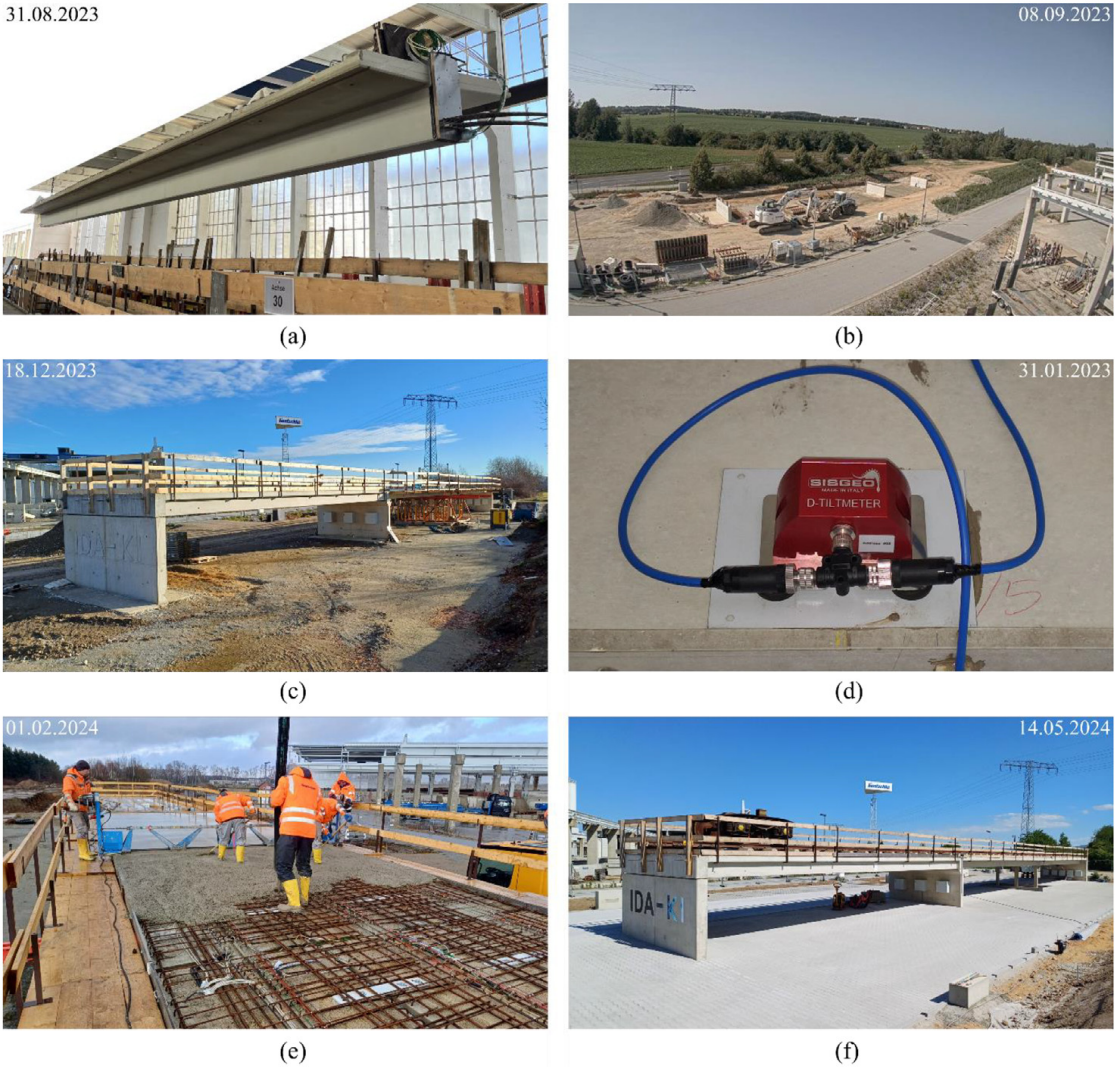
Construction process of the openLAB: (a) pretensioning of PEs and stripping the formwork; (b) fabrication of the substructures; (c) assembled PEs and temporary scaffolding in axis 20; (d) start of the measurements; (e) pouring of the in-situ cast concrete deck; (f) completed bridge with installed load vehicle (Photos: Hentschke Bau GmbH, Max Herbers, Robert Röder).

Understanding the monitoring data requires consideration of the different construction stages. Fig. 6 provides visual impressions of the construction process, while more detailed information on the construction timeline is presented in Table 6.

**Table 6:** Construction history. For an overview of the structure and axis layout, see Fig. 1.

Date	Location	Axis/PE No.	Work description
2023-07-12	On site	10	Concreting clean layer
2023-07-13	On site	20	Concreting clean layer
2023-07-20	On site	30	Concreting clean layer
2023-07-20	On site	10	Concreting foundation
2023-08-09	On site	20	Concreting foundation
2023-08-14	On site	30	Concreting foundation
2023-08-23	Precast Plant	PE 2.2	Concreting
2023-08-24	Precast Plant	PE 2.2	Pretensioning and stripping the formwork
2023-08-30	On site	30	Concreting pier wall
2023-08-30	Precast Plant	PE 2.3	Concreting
2023-08-31	Precast Plant	PE 2.3	Pretensioning and stripping the formwork
2023-09-04	On site	10	Concreting abutment wall
2023-09-06	Precast Plant	PE 1.3	Concreting
2023-09-12	Precast Plant	PE 1.3	Pretensioning and stripping the formwork
2023-09-13	Precast Plant	PE 1.2	Concreting
2023-09-19	Precast Plant	PE 1.2	Pretensioning and stripping the formwork
2023-09-20	Precast Plant	PE 1.1	Concreting 1st section near A 20
2023-09-26	Precast Plant	PE 1.1	Partial posttensioning of 1st section near A 20 (tendon with subsequent bonding), concreting 2nd section near A 10
2023-09-27	On site	20	Concreting columns
2023-10-02	Precast Plant	PE 1.1	Partial posttensioning 2nd section near A 10 (tendon with subsequent bonding), Pretensioning part 1 + 2 with immediate bonding
2023-10-05	Precast Plant	PE 2.1	Concreting
2023-10-06	Precast Plant	PE 2.1	Partial posttensioning, pretensioning, stripping the formwork
2023-10-12	Precast Plant	PE 1.1+2.1	Full posttensioning
2023-10-13	Precast Plant	PE 1.1+2.1	Grouting
2023-11-27	On site	Span 1 and 2	Assembly of all PE
**2024-01-31**	**On site**	**Span 1 and 2**	**Installation of the SHM system and start of the measurements**
2024-02-01	On site	Span 1 and 2	Concreting in-situ concrete layer
2024-02-22	On site	Span 1 and 2	Lowering temporary scaffolding
2024-03-18	On site	Span 1 to 3	Installation of tracks for load vehicle
2024-04-19	On site	Span 1 to 3	Placing the load vehicle
2024-04-22	On site	Span 1 to 3	Installation of the load vehicle (load = 4.1 t)
2024-06-27	On site	Span 1 to 3	Mounting frame (additional load) on load vehicle (load = 4.9 t)

### 4.3 Changes in the Static System

**Fig. 7:**
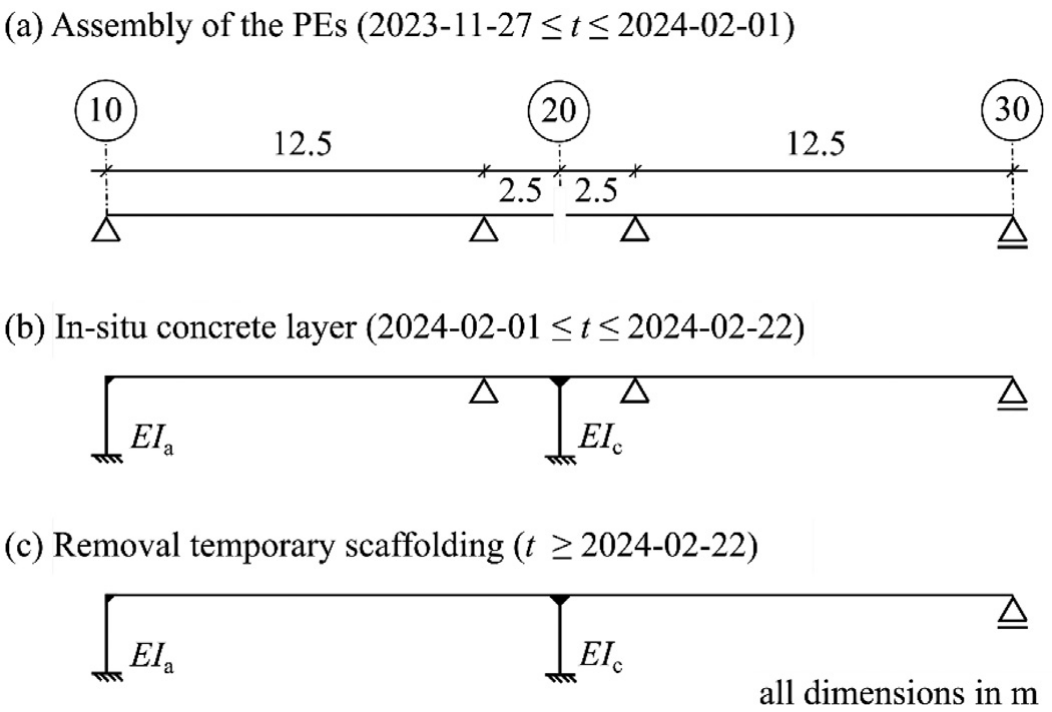
Evolution of the static system of span 1 and 2 during the construction process: (a) assembly of the PEs; (b) pouring in-situ concrete; (c) removal of the temporary scaffolding (Graphic: Max Herbers).

The static system changed several times during construction. Initially, when the PEs were assembled, they behaved as simply supported girders with a small cantilever due to the temporary scaffolding near axis 20, Fig. 7 (a). With the pouring of the in-situ concrete deck and progressive hydration, the system merges into a frame structure, Fig. 7 (b). In axes 10 and 20, the PEs are monolithically connected to the substructures, whereas in axis 30, the PE are supported by elastomeric pads. With the removal of the scaffolding, the final system is in place, Fig. 7(c). Due to the high stiffness of the abutment wall in axis 10 compared to the slender columns in axis 20, the fixed point can be assumed to be in axis 10 (*EI*_a_ ≫ *EI*_c_).

The evolution of the eigenfrequencies of spans 1 and 2 during construction and the first 72 days after pouring the in-situ concrete layer can be found in [4]. In [17] it is shown that the bending eigenfrequency correlates with the concrete’s compressive strength and increases as hydration progresses. The corresponding monitoring data are freely available in [18].

### 4.4 Material Properties

All steel reinforcement bars are standard ribbed bars, conforming to DIN EN 1992-2, DIN EN 10080, and DIN EN 13670 with DIN 1045-3, with a characteristic yield strength of *f*_yk_ = 500 N/mm². By default, the prestressing strands are 7-wire strands with a diameter of *d* = 12.5 mm and a cross-sectional area of *A*_p_ = 93 mm². In contrast, the strands in the tendon of PE 1.1 and PE 2.1 have a diameter of *d* = 15.7 mm and a cross-sectional area of *A*_p_ = 150 mm². Prestressing steel is classified as St 1660/1860. No specific material test data are available for reinforcement and prestressing strands, as this is not required by DIN EN 13670, DIN 1045-3, and DIN EN 13369.

Standard normal-strength concrete in accordance with DIN EN 206-1/DIN 1045-2 was used for both the PEs and the in-situ cast components, as follows:

• C30/37 for substructures (foundations, abutment walls, and the pier wall at axis 30) and some elements of the superstructure (PE 1.1, PE 1.2, PE 1.3, and the in-situ cast concrete deck of spans 1 and 2)
• C50/60 for piers at axis 20 and for all PEs of spans 2 and 3

The following data are available for concrete, mostly due to requirements of DIN EN 13670, DIN 1045-3, and DIN EN 13369:

• Compressive strength according to DIN EN 12390-3 at 150 mm cubes (DIN EN 12390-1) at concrete ages of 1, 2, 7 and 28 days from initial testing,
• Youngs modulus according to DIN EN 12390-13 at concrete age of 28 days from initial testing,
• Density according to DIN EN 12390-7 from initial testing,
• Slump according to DIN EN 12350-2 at 5 min and 45 min from initial testing,
• Compressive strength at the time of prestressing, measured using the maturity method according to NEN 5970:2001 during production control,
• Compressive strength DIN EN 12390-3 at 28 d, measured for each cast during production control, see Table 7.

**Table 7:** Concrete compressive strength (span 1 and 2) at 28 days from production control according to DIN EN 12390-3

Element	planned concrete strength class	date of production	cube 1 *f*_c1_ in N/mm²	cube 2 *f*_c2_ in N/mm	cube 3 *f*_c3_ in N/mm	mean *f*_cm_ in N/mm²
PE2.2	C50/60	2023-08-23	78.5	78.7	77.9	78.4
PE2.3	C50/60	2023-08-30	81.6	81.6	78.7	80.6
PE1.3	C25/30	2023-09-06	49.8	50.2	51.4	50.5
PE1.2	C25/30	2023-09-13	49.8	48.6	49.0	49.1
PE1.1/Section 1	C25/30	2023-09-20	49.9	48.7	47.7	48.8
PE1.1/Section 2	C25/30	2023-09-26	46.3	44.9	43.4	44.9
PE2.1	C50/60	2023-10-05	76.4	78.8	79.4	78.2
Pier axis 30	C30/37	2023-08-30	66.0	64.3	64.4	64.9
Pier axis 10	C30/37	2023-09-04	68.2	68.9	70.7	69.3
Piers axis 20	C50/60	2023-09-27	61.4	64.1	68.3	64.6
In-situ concrete deck span 1+2	C30/37	2024-02-01	51.6	52.4	52.7	52.2

Detailed information on concrete and reinforcement are documented at the formwork and reinforcement drawings, available in the dataset or via EPLASS InfoClient, see [15]. Further data can possibly made available upon request, e.g., concrete mix designs.

### 4.5 Sensor Layout

The electrical SHM system includes sensors installed on spans 1 and 2 of the bridge. Sensor locations are documented in the BIM models, and technical specifications are available in the datasheets – both provided in the supplementary materials accompanying the dataset. Installation was completed in late January 2024, with measurements starting on January 31, just before the in-situ slab layer was poured, see Table 6.

**Fig. 8:**
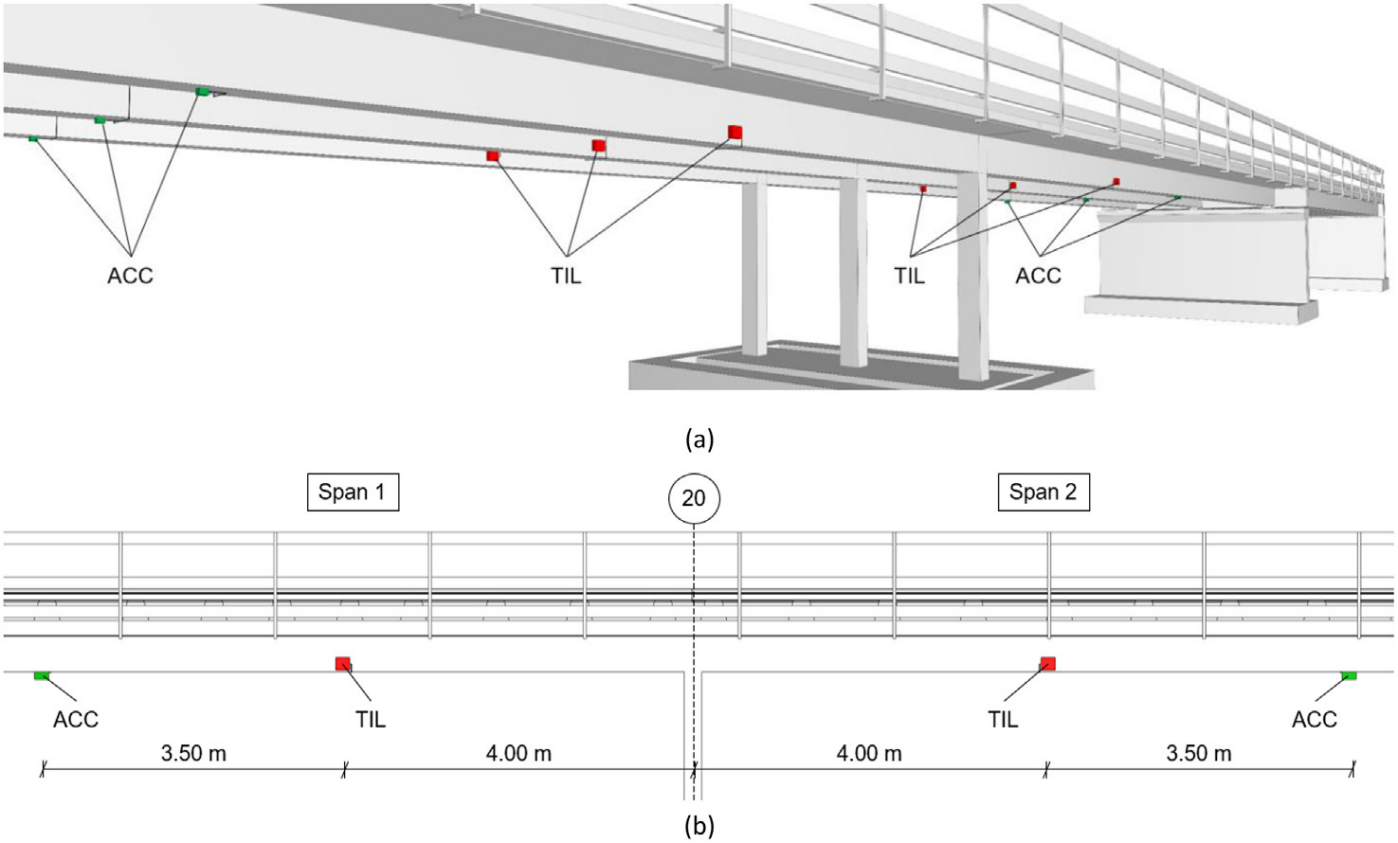
Sensor layout for acceleration and tilt measurements: (a) 3D visualization (b) Elevation view.

Accelerometers (PCB 393A03) are mounted at midspan of both spans, with one sensor per girder, as shown in Fig. 8. These locations were chosen because midspan sections are expected to exhibit the highest vibration amplitudes. The use of six accelerometers enables the identification of several mode shapes.

The digital tiltmeters (Sisgeo 0S542HD0502) are also installed on both spans, with one sensor per girder, resulting in a total of six sensors. The tiltmeters are positioned 4 m from axis 20, approximately at the location of zero bending moment under self-weight, where the highest tilt is expected.

The climate sensors (Thies Clima 1.1005.54.773, Kipp & Zonen SP Lite2) are mounted above the bridge in axis 10.

In addition to the conventional sensors, a comprehensive network of DFOS has been installed within the PEs and piers. This allows condition monitoring from the moment the concrete is poured [4]. However, the DFOS monitoring concept and data will be the subject of a separate publication.

### 4.6 Test Concept

**Fig. 9:**
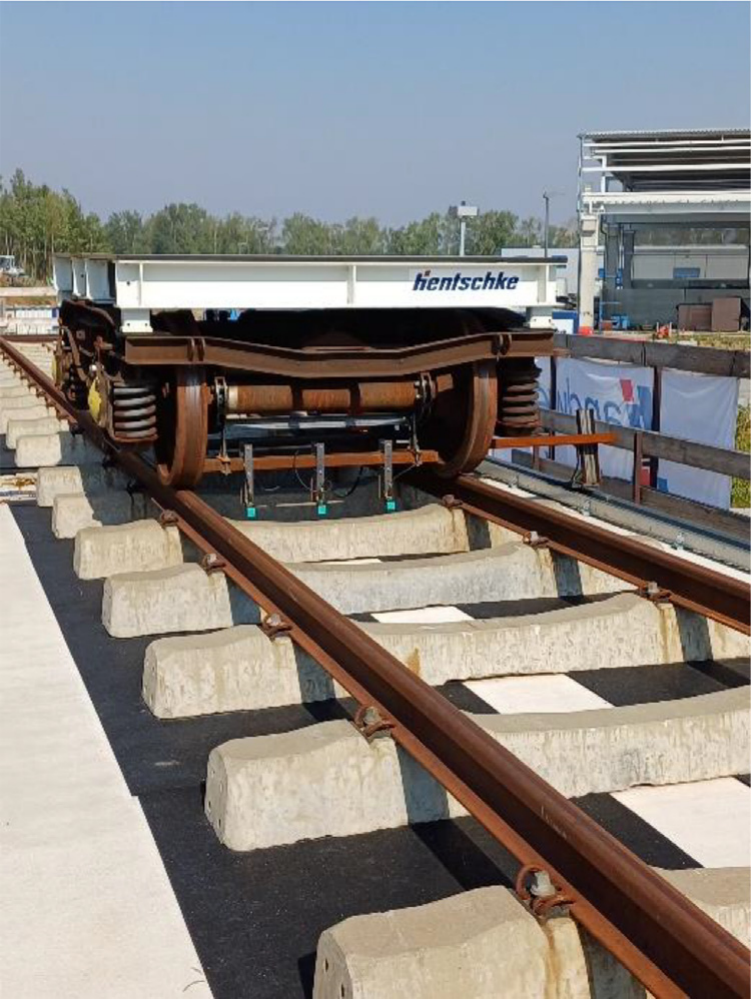
Rail guided load vehicle (Photo: Hentschke Bau GmbH).

After the completion of the bridge, the undamaged reference condition is recorded over a one-year period under varying climatic conditions and occasional traffic loads, referred to as the *reference phase*. To simulate traffic loads, a rail-guided vehicle, as shown in Fig. 9, was driven over the bridge 30 to 40 times per day on a designated test day each month. Additionally, to analyze the dynamic behavior across different frequency ranges, the bridge was excited using a shaker.

**Fig. 10:**
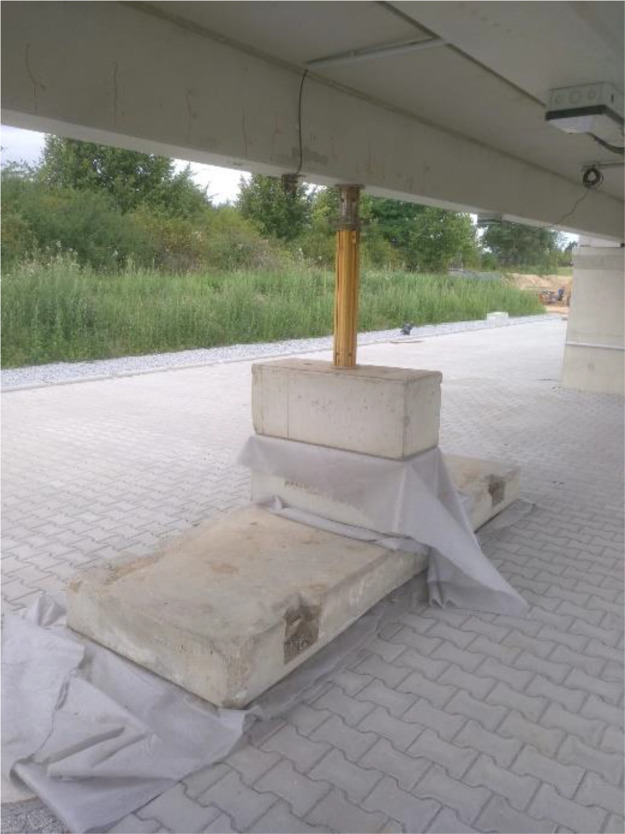
Installed prop in the middle of PE 2.1 to restrict downward deformations (Photo: Hentschke Bau GmbH).

To assess whether the existing evaluation methods can detect structural changes, a non-destructive test was conducted from July 22 to August 2, 2024. As shown in Fig. 10, a temporary prop was placed at midspan of PE 2.1 to restrict downward deformation, thereby altering the bridge’s deformation behavior. This intervention allows researchers to observe how changes in structural conditions are reflected in the monitoring data.

Following the reference phase, the bridge will be subjected to load tests up to a severely damaged state. The loads will be applied up to ULS using counterweights, a load traverse, and hydraulic jacks. Additional structural damage, e.g., to the tendons, will be introduced. Furthermore, sensor faults will be intentionally induced to evaluate the performance of the SHM system and the effectiveness of the evaluation algorithms in detecting anomalies and distinguishing between sensor faults and actual structural damage.

The load tests will be discussed in detail in subsequent publications. A schedule of the conducted and planned load tests is available via the EPLASS InfoClient [15].

## Limitations

The acceleration measurements faced several challenges:

• Initially, a 5-min acceleration sample was recorded every hour. However, under ambient excitation, the bridge exhibits minimal vibration activity. As a result, the samples were unsuitable for modal analysis. Consequently, a trigger mechanism was implemented to record acceleration data only when amplitudes exceeded a predefined threshold.
• A malfunctioning cable resulted in data loss for the sensor G_ACCZ_PE21_BU0750_0 until July 26, 2024.
• The recorded signals display frequency components around 50 Hz and its harmonics, likely caused by the AC/DC power supply of the measurement system.
• In particular, the sensor G_ACCZ_PE12_BU0750_0 shows some sudden spikes in its time-domain signal. Although the cause of this behavior remains unknown, its impact on frequency-domain analyses is assumed to be negligible.

Measurements from the tiltmeters and environmental sensors demonstrated reliable performance throughout the monitoring period. However, during June and July, connection issues with the Network Time Protocol (NTP) server led to potential time synchronization errors, resulting in data timestamps deviating by a few minutes.

Researchers intending to use the tiltmeter data for anomaly detection should be aware of occasional test vehicle crossings outside the scheduled monthly load tests. Depending on the chosen detection method, these unscheduled crossings may be classified as anomalies. Therefore, it is advisable to implement a filtering mechanism to exclude these events from the training dataset to improve the accuracy of anomaly detection models.

## Ethics Statement

The authors confirm that they have read and follow the ethical requirements for publication in Data in Brief and that the current work does not involve human subjects, animal experiments, or any data collected from social media platforms.

## CRediT authorship contribution statement

**Andreas Jansen:** Data curation, Methodology, Writing – original draft, Visualization. **Max Herbers:** Conceptualization, Methodology, Writing – original draft, Visualization, Project administration, Funding acquisition. **Bertram Richter:** Data curation, Writing – review & editing. **Maria Walker:** Writing – review & editing. **Frank Jesse:** Methodology, Writing – review & editing, Funding acquisition. **Steffen Marx:** Resources, Supervision, Funding acquisition.

## Data Availability

OPARAMonitoring Data of the openLAB Research Bridge (2024-02-01 to 2024-10-31) and building information (Original data)

## Appendix Supplementary materials

Image, application 1
