# Supplementary material for: Monitoring data of the openLAB research bridge – Part 1: Reference condition
Source: Data Brief. 2025 May 7;60:111624. doi: 10.1016/j.dib.2025.111624 (PMC12145563; doi:10.1016/j.dib.2025.111624)
Supplement: Supplementary file 1 [file mmc1.pdf]

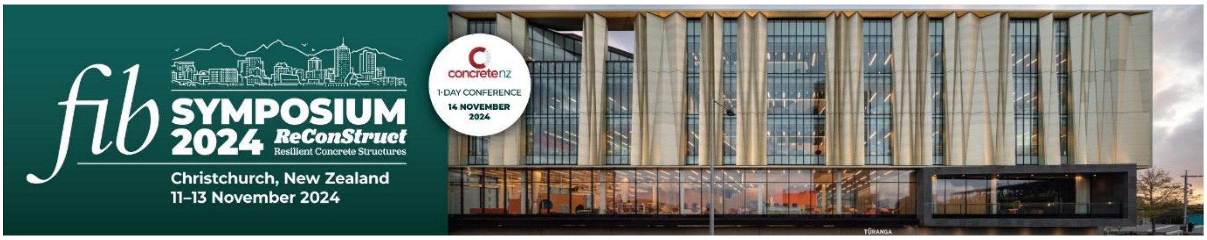

openLAB – A large-scale demonstrator for advancing digital twin developments of bridges

Max Herbers[1*], Bertram Richter[1], Jan-Hauke Bartels[1], Thamer Al-Zuriqat[2], Kay Smarsly[2], Steffen Marx[1]

[1] Institute of Concrete Structures, Dresden University of Technology, Germany
*max.herbers@tu-dresden.de
[2] Institute of Digital and Autonomous Construction, Hamburg University of Technology, Germany

Abstract. Digital twins are being used for predictive maintenance of civil infrastructure. The real-time capabilities of digital twins are typically achieved through metrological monitoring of real objects, the “physical twins”. Monitoring processes of civil infrastructure usually generate large amounts of data, which can only be exploited if the monitoring data is automatically evaluated and assessed. Due to a lack of monitoring data that reflects actual structural damage (e.g., on bridges), existing evaluation methods are not sufficiently validated. Within the IDA-KI research project, a 45 m long, three-span, prestressed concrete (PC) research bridge, the “openLAB” bridge has been built and equipped with a monitoring system as a case study for investigating actual damage scenarios. Parts of the measurement technology, such as distributed fiber optic sensors, have been installed before pouring the concrete. In the first year after completion, the undamaged reference condition of the bridge is measured under climate and traffic impact. Next, load tests up to the state of severe damage will be carried out to create a database containing monitoring data corresponding to structural damage as well as to sensor faults of the monitoring system. This paper presents the concept for the IDA-KI research project, and first results of a system identification during construction and after completion of the PC bridge. Furthermore, the openLAB bridge is introduced with its structural features and the monitoring system. In the paper, methods for automated data analysis and sensor fault diagnosis are proposed and will be tested later using the monitoring data recorded from the openLAB bridge.

Keywords: Digital twin, civil infrastructure, openLAB bridge, structural health monitoring, automated data analysis.

1 Introduction

Real-world monitoring data is fundamental for scientific progress and societal development. The collection and analysis of data from rare events facilitates a better understanding of complex relationships and enhances resilience to future challenges. Data sets of outstanding importance are, for example, (i) the Nuclear Power Plant Accidents Database, which collects information on reactor accidents and incidents in nuclear power plants, (ii) the National Transportation Safety Board (NTSB) Aviation Accident Database, which is a collection of aircraft accidents and incidents, and (iii) the National Aeronautics and Space Administration (NASA) Earth Observing System Data and Information System (EOSDIS), which collects data on environmental phenomena, such as climate change and natural disasters. Real-world monitoring data is also essential for developing digital twins of civil infrastructure and for detecting structural damage or changes in load-bearing behavior. The monitoring data can be used as a basis for

the development of structural evaluation methods, so that large amounts of data can be efficiently processed and aggregated into tangible information on the condition of structures [1]. Data sets of events of interest that include both damage to real structures and sensor faults under well-defined conditions are rare, but crucial for validating the structural evaluation methods.

The IDA-KI project [2] aims to develop methods for automated evaluation and assessment of monitoring data and to validate the structural evaluation methods using real-world monitoring data that include sensor faults and features from actual structural damage. To this end, a 45 m long prestressed concrete bridge is built as a case study and equipped with an extensive structural health monitoring (SHM) system, which provides information on the condition of the structure from the beginning of concrete casting and throughout the operational phase of the bridge, which involves inflicting damage until a “severely damaged state” has been reached. The bridge is used as an object for scientific exchange and joint experimental campaigns, and it is therefore referred to as “openLAB” bridge. The IDA-KI research project consists of two phases, a one-year reference phase and a load-testing phase. Upon completion of construction, the reference phase begins, during which the structure is exposed to only moderate, simulated traffic loads and climatic influences. Thereafter, load tests are conducted in the load-testing phase up to the severely damaged state. Typical structural deficiencies of prestressed concrete (PC) bridges have been induced in some of the precast elements (PEs) to create a database with specific features in monitoring data representing different damage mechanisms. In addition, sensor faults are induced into the SHM system to build a basis for developing and validating structural evaluation methods for reliably differentiating between sensor faults and structural damage. All relevant information about the openLAB bridge is provided via a digital twin, which combines different specialized building information models, e.g., for structural inspections, diagnostic investigations and SHM, in a coordination model [3]. Information aggregation about the condition of the openLAB bridge enables intuitive asset management.

This paper gives an overview of the IDA-KI research project, given in Section 2, including an introduction to the openLAB bridge, its structural features, the SHM system, and the test concept. Methods of data evaluation for different measurement technologies are discussed in Section 3, and results of the eigenfrequency determination during the construction process of the bridge are presented in Section 4. Finally, conclusions drawn from the results are provided, along with potential future work.

2 The openLAB bridge

The openLAB bridge – built in Bautzen, Germany – is a three-span, PC bridge, 45 m long and 4.5 m wide. As illustrated in Fig. 1, the bridge employs a wide range of common construction types, and each span of the has its own research focus.

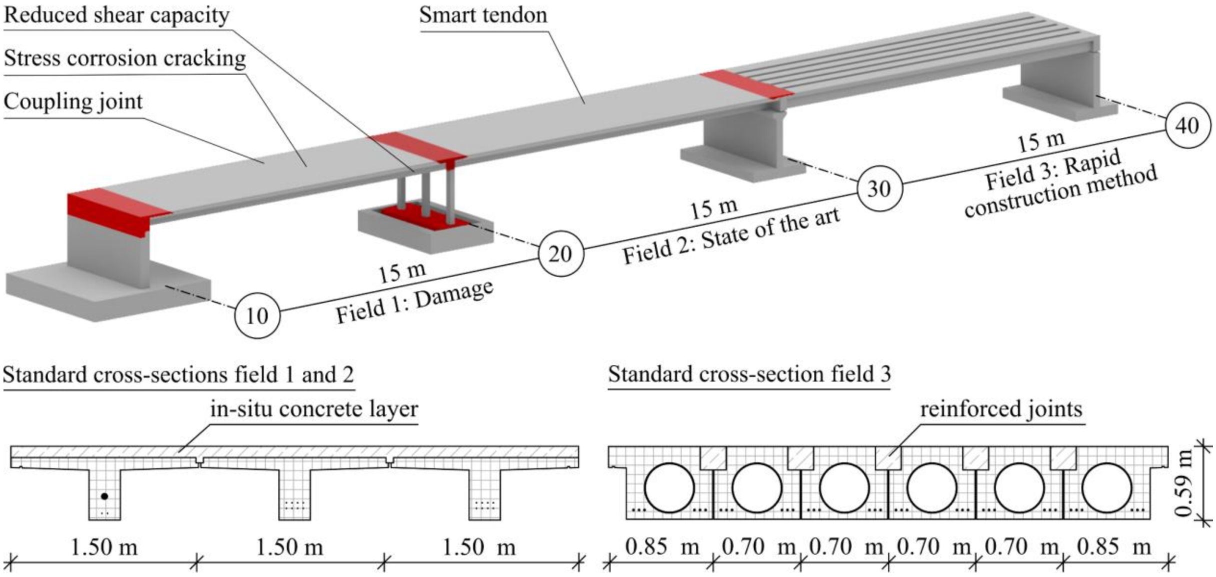

Fig. 1. Illustration of the openLAB bridge including the standard cross-sections (Graphic: Fabian Collin, Max Herbers).

Spans 1 and 2 consist of three prestressed PEs each, with T-shaped cross-sections, overlaid with cast-in-place concrete. Span 1 contains typical structural deficiencies of early prestressed concrete constructions, such as coupling joint problems, stress corrosion cracking, and areas of reduced shear capacity. Gravel pockets and blowholes are also present. Span 2 is built using state-of-the-art technology. One PE is post-tensioned with a so-called "smart tendon", which integrates distributed fiber optic sensors (DFOS) into the tendon strand to measure the strain distribution and evolution [4]. The goal is to detect damage to the tendon and the structure at an early stage. Span 3 consist of slab built employing a rapid construction system without cast-in-place concrete, which is fully loadable almost immediately after installation. Cylindrical hollow steel bodies have been integrated into the PEs to reduce the dead weight. Load distribution in the transverse direction is achieved by reinforced joints, which are cast in place.

By default, the PEs are prestressed with strands, with immediate bond. Only two PEs, representing coupling joint problem or including the smart tendon, are post-tensioned. Structural features, highlighted in red in Fig. 1, include (i) monolithic connections between superstructure and substructures in axes 10 and 20, (ii) a movable foundation in axis 20, and (iii) a connection with a cement-based ultra-high-performance fiber-reinforced cementitious composite material (UHPFRC) in axis 30, which ensures water tightness and fast trafficability [5]. However, the connection will be produced at a later stage (after the first load tests), so the spans 2 and 3 are currently statically independent.

To achieve loading conditions up to the ultimate limit state (ULS) with reasonable testing effort, only 25% of load model 1 according to DIN EN 1991-2 was considered for the design. This results in a rather high slenderness of l/d = 15/0.59 m ≈ 25, where l is the span length, and d is the cross-section height. Fig. 2 shows the openLAB bridge shortly before completion in April 2024, as well as the rail installation on the bridge deck for a load vehicle.

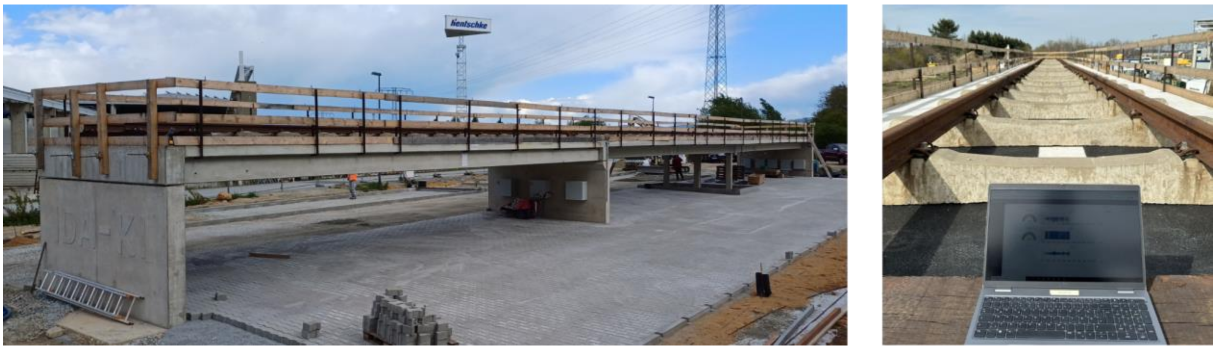

Fig. 2. The openLAB bridge shortly before construction completion in April 2024 (left); Installation of rails for a load vehicle and first measurements (right) (Photos: Hentschke Bau GmbH, Thamer Al-Zuriqat).

2.1 Structural health monitoring system

Parts of the SHM system, such as DFOS and temperature sensors, has already been installed in the formwork prior to concreting to provide information about the initial condition of the PEs (e.g., hydration process, early shrinkage, prestressing). The "early-age" monitoring from the beginning of the existence of the structure has a significant advantage over reactive or event-driven monitoring applications, as the undamaged reference condition is known. With the SHM system, a distinction is made between global and local measurements. Acceleration, inclination, and displacement measurements are used for the assessment of the global structural behavior. The load tests up to the severely damaged state are carried out to analyze the extent to which damage of different type and severity affects the modal parameters or the deformation shape. However, the most common types of damage are local phenomena and have a low effect on the global structural behavior [6]. The SHM system is therefore complemented by local measurements, which are primarily arranged in the areas where deficiencies are induced. The combination of global and local measurements is designed to enable reliable damage detection. In what follows, the conventional metrology, employed for both global and local measurements, as well as the DFOS technology are discussed. It is foreseen that the SHM system is expanded with additional third-party measurement technology to evaluate the potential of different sensor types in detecting structural defects during the load-testing phase.

Conventional metrology for continuous monitoring

During the reference phase, SHM focuses on spans 1 and 2. As shown in Fig. 3(a), an accelerometer and an inclinometer are installed on each PE. Accelerometers are permanently mounted on magnetic adapter plates at the mid span, Fig. 3(b). During the construction of the bridge, additional accelerometers were glued next to the permanent sensors, cf. Section 4. Inclinometers are bolted to the web in the area of the zero moment crossings (M_0) near axis 20, Fig. 3(c). A laser distance sensor measures the length changes of the superstructure. Additional sensors are employed to measure environmental conditions, i.e., air temperature, relative humidity, solar radiation, and precipitation.

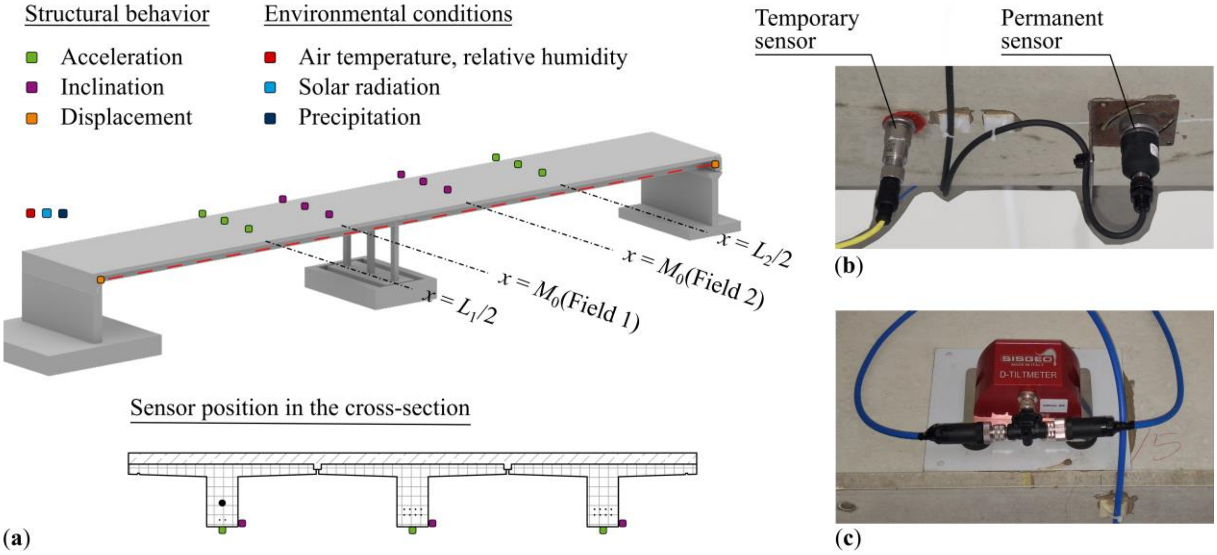

Fig. 3. Overview of the conventional metrology: (**a**) Sensor placement; (**b**) comparison of different accelerometers; (**c**) inclinometer (Graphic: Fabian Collin, Max Herbers).

Strain gages (SGs), linear displacement transducers, and temperature sensors are used for local measurements. SGs are glued on selected rebars and are taken as reference for comparing measurements from the DFOS. Five temperature sensors are installed in each PE at the level of the lower reinforcement. Additional temperature sensors are installed in the cast-in-place concrete. The monitoring started before the in-situ concrete is poured. The monitoring data can be viewed and tracked trough a web-based graphical user interface called "iris", which allows users to set thresholds that trigger alerts. To diagnose sensor faults, the evaluation procedures described in Section 3.1 are implemented. Detailed information about the SHM system can be obtained from a specialized building information model provided by a common data environment [3], referred to as "SHM model". External users can access the model via the IDA-KI project website [7].

Distributed fiber optic sensing

The conventional metrology presented above is extended by periodic distributed fiber optic measurements. The measurements are taken at specific times, e.g., during prestressing or loading tests. In addition, monthly measurements are taken only under deadweight and climatic effects. A DFOS network with a total length of more than one kilometer is installed in the bridge, as illustrated in Fig. 4(a). DFOS embedded in the concrete enables monitoring from the beginning of the existence of the structure, which makes it possible to assess the initial integrity. Furthermore, the DFOS can be utilized to monitor changes in structural behavior over the life time of the bridge. With Rayleigh-based interrogators, DFOS with a length of up to 100 m and a spatial resolution in the millimeter range can be measured. This makes it possible to simultaneously capture local effects, such as damage to tendons or cracks, and to gain information on global structural behavior.

Different acquisition units, sensor types and installation techniques for the DFOS technology are compared for ensuring reliable monitoring with reasonable effort. Preliminary tests are carried out to select suitable sensor types for the openLAB bridge [8]. For real-world construction applications, robust DFOS are recommended to prevent damage during installation and concrete pouring. With robust

DFOS, the optical fiber in the primary coating is surrounded by additional protective layers of plastic or metal, reducing the sensitivity [9]. For example, slippage may occur within the DFOS layers, attenuating the strain curve, which might make the interpretation of the measurement data challenging. Experimental investigations have shown that robust DFOS with a monolithic cross-section exhibit an optimized strain transfer and can therefore detect small local strain changes.

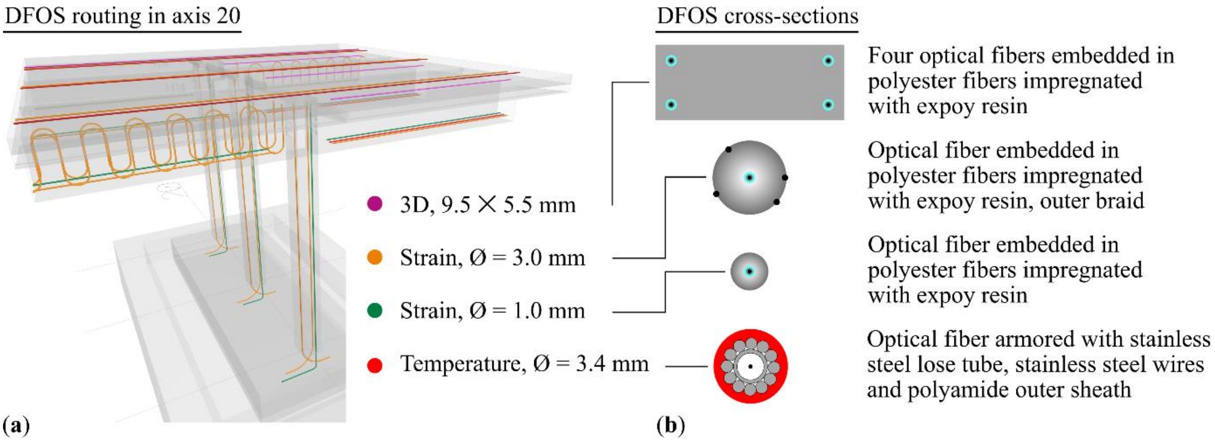

Fig. 4. The DFOS are integrated into the structure for distributed strain, deformation, and temperature measurements: (**a**) DFOS routing in axis 20; (**b**) cross-sections of applied DFOS types (not to scale) (Graphic: Max Herbers, Fabian Collin).

Depending on the measurement task, different DFOS types are used, as illustrated Fig. 4(b). To measure strain in the reinforcement, DFOS with a smooth surface and monolithic cross-section (Ø = 1 mm) are glued to the rebars using a 2-component epoxy adhesive. DFOS with a diameter of 3 mm and a textured outer surface for optimized bonding properties are also directly embedded in the concrete matrix. To secure the position of the DFOS during concreting, the DOFS are attached to the stirrups using cable ties. Since the frequency shift in Rayleigh-based fiber optic measurements results from both mechanical strain and temperature changes, temperature effects must be compensated in strain measurements. Several methods for temperature compensation are being investigated within the project: (i) measurements with special mechanically decoupled DFOS, (ii) local temperature measurements along the DFOS axis, and (iii) use of acquisition units that evaluate Raman backscattering, which is exclusively sensitive to temperature effects [10]. A special feature is the employed 3D-DFOS, in which four optical fibers are incorporated into the cross-section [11]. The curvature can be determined from the strain differences and the distance measured between the individual fibers. Thus, the changes in the deformation figure in both the vertical and horizontal directions can be determined by integration.

By default, two DFOS are installed at the level of the lower reinforcement of each PE – one bonded to the reinforcement and the other embedded in the concrete matrix. Further DFOS are arranged, for example, in the region with reduced shear-force load-bearing capacity, near the prestressing anchor, in the monolithic connections between superstructure and columns (axis 20) and in the in-situ concrete layer. To accurately locate anomalies when interpreting the monitoring data, the DFOS routing is carefully surveyed by hand and locally referenced with a precision in the order of centimeters. All DFOS are georeferenced with semantic information in the SHM model. The reference state (called "fingerprint") of the DFOS is set prior to concreting. A tare measurement after compaction is recommended to compensate for strain imposed by concreting. The production processes of selected PEs are measured continuously during concrete hardening and prestressing to capture the onset of hydration heat, early-age shrinkage and prestressing strain.

2.2 Test concept

After completion of the bridge, the undamaged reference condition is first measured over a one-year period under climatic conditions and traffic loads (reference phase). To simulate traffic loads, a rail-guided vehicle is ballasted and driven over the bridge several times a month. To analyze the dynamic behavior in different frequency ranges, the bridge is also excited with a shaker.

After the reference phase, the bridge will undergo load testing up to the severely damage state. The force will be applied up to the ULS using counterweights, a load traverse and hydraulic jacks. Additional structural damage, e.g., to the tendons, will be introduced and sensor faults will be intentionally induced to test the ability of the SHM system and the evaluation algorithms to detect anomalies and differentiate between sensor faults and structural damage.

3 Automated data evaluation

Automated data analysis is used to extract valuable information about the condition of the structure from the raw monitoring data with minimal latency. Conventional metrology (e.g., SG, inclinometers, and accelerometers) provides monitoring data as measurement time series, consisting of time stamps associated with physical quantities being measured. In Section 3.1, an approach is presented that enables fully automated sensor fault diagnosis (FD) in SHM systems using the measurement time series of the conventional metrology. Next, an open-source framework for automated data processing and information extraction from DFOS strain measurements is presented in Section 3.2.

3.1 Sensor fault diagnosis for conventional metrology

Sensor faults, typically caused by hardware or software malfunctions, power outage, environmental impacts or signal interferences [12], may affect the reliability and performance of SHM systems. Sensor FD approaches for SHM systems have been proposed primarily based on physical or analytical redundancy [13]. In physical redundancy approaches, fault detection relies on comparing monitoring data recorded by redundant sensors of the SHM systems. The high cost, power consumption, and maintenance required for physical redundancy approaches have been the primary motivations for employing so called “analytical redundancy” [1]. Analytical redundancy for sensor FD builds upon mathematical models, such as finite element models or machine learning techniques, for mapping relationships between monitoring data from different sensors, leveraging the inherent redundancy present in the monitoring data [14]. In general, sensor FD comprises four steps as outlined in [15]. First, *fault detection* involves recognizing any abnormal operation within the system. Second, *fault isolation* entails precisely specifying the location of the fault. Third, *fault identification* focuses on determining the specific type or nature of the fault. Finally, *fault accommodation* aims to compensate for the adverse effects caused by the fault.

The IDA-KI project introduces a decentralized sensor FD approach using a hybrid machine learning method that combines regression [16] and classification [17] techniques. While current FD approaches operate in a centralized manner, in which a single fault may lead to a complete system failure, in this project, the decentralized sensor FD approach is embedded into tailor-made wireless sensor nodes of the SHM system, reducing the risk of single-failure points. The FD process is described as follows: In an initialization phase, monitoring data from different sensors undergo a correlation analysis to define sets of “correlated sensors”. Data recorded from correlated sensors is used for training artificial neural network (ANN) regression models and long short-term memory (LSTM) classification models on a central computer of the SHM system. Then, the ANN and LSTM models are embedded into the wireless sensor nodes of the SHM system with each ANN model producing virtual outputs for one sensor of the SHM system using data from correlated (typically neighboring) sensors. If residuals between actual monitoring data and the virtual outputs of the ANN model exceed a fault detection threshold [18], a fault is detected and isolated. Isolated faults are then fed to the LSTM models to specify the type and nature of the fault. Finally, fault accommodation is performed by replacing faulty monitoring data with virtual outputs of the ANN models. Fig. 5 presents the decentralized sensor FD approach introduced in the project, which has been validated (i) by simulating and artificially injecting faults into monitoring data [16], (ii) performing laboratory tests using a shake table system [19], and (iii) using data from a real-world SHM system [20]. The results in the aforementioned references demonstrate the suitability of the FD approach for ensuring the reliability and performance of SHM systems.

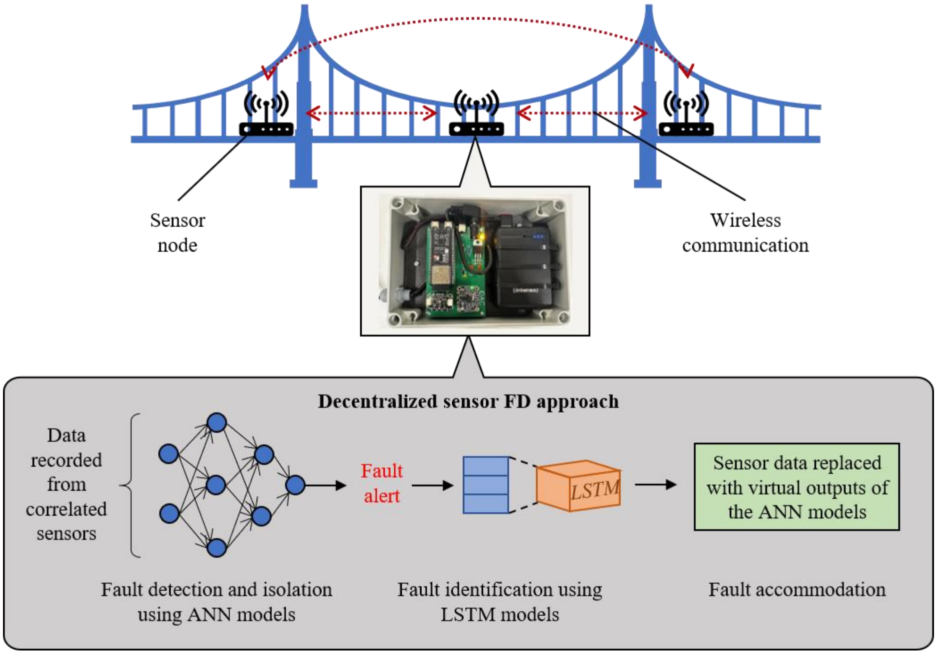

Fig. 5. Schematic representation of the decentralized sensor FD approach (Graphic: Thamer Al-Zuriqat).

3.2 Evaluation of the DFOS data

By contrast to conventional metrology, whose sensors provide time series for specific physical quantities, DFOS provide time series of arrays (e.g., distributed strain curves), that allow investigating a wide variety of research questions. The distributed character is a decisive advantage of DFOS over conventional metrology, but the amount of data generated is orders of magnitude higher. Hence, manual evaluation is impractical, and automation becomes a necessity [21, 22].

The free open-source software framework "fosanalysis" [23] is developed to streamline the data analysis process for the DFOS data from reading measurement files to selecting, preprocessing and evaluating the data [21]. The framework structure is designed in a modular fashion to facilitate maintainability, extensibility, and flexibility. Complex workflows are composed from simpler tasks, enabling fine-grained, but comprehensible control of the workflow. The software framework "fosanalysis" implements automated crack monitoring, including determination of the crack location and crack width. The workflow is as follows: First, the measuring segment of interest is extracted from the strain profile. Second, the strain data is preprocessed to remove measurement disturbances (noise, strain reading anomalies and dropouts) [24]. Third, cracks are detected by a peak finding algorithm and, fourth, the integration lengths are determined, which serve as the limits for the following numerical integration. Fifth, the influence of tension stiffening is compensated according to [25, 26] and, finally, the crack width is determined by numerical integration.

4 First results: System identification during construction

To analyze the dynamic behavior of the bridge and the influence resulting from system changes and concrete hardening, initial acceleration measurements during construction were taken using uniaxial integrated electronic piezoelectric (IEPE) sensors, as shown in Fig. 3. Preliminary experimental investigations with the IEPE sensors show that particularly low eigenfrequencies and less noisy measurements can be recorded, as compared to micro-electro-mechanical system (MEMS) sensors [27].

The first bending eigenfrequency of the superstructure is calculated over time from the acceleration measurements using the Fast Fourier Transformation (FFT). The excitation of the bridge is ambient, i.e., caused by external environmental factors, mainly consisting of heavy trucks driving on a federal highway in the direct vicinity of the bridge. Time windows in the acceleration measurements with significant amplitudes have manually been detected in the time domain, and the respective windows have been transformed into the frequency domain using the FFT. The measurements have been performed

continuously over a period of 82 days from January 22, 2024 to April 12, 2024 with a sampling rate of 200 Hz. Fig. 6 shows the results of the bending eigenfrequency during construction, with the first bending eigenfrequency at about 6.4 Hz.

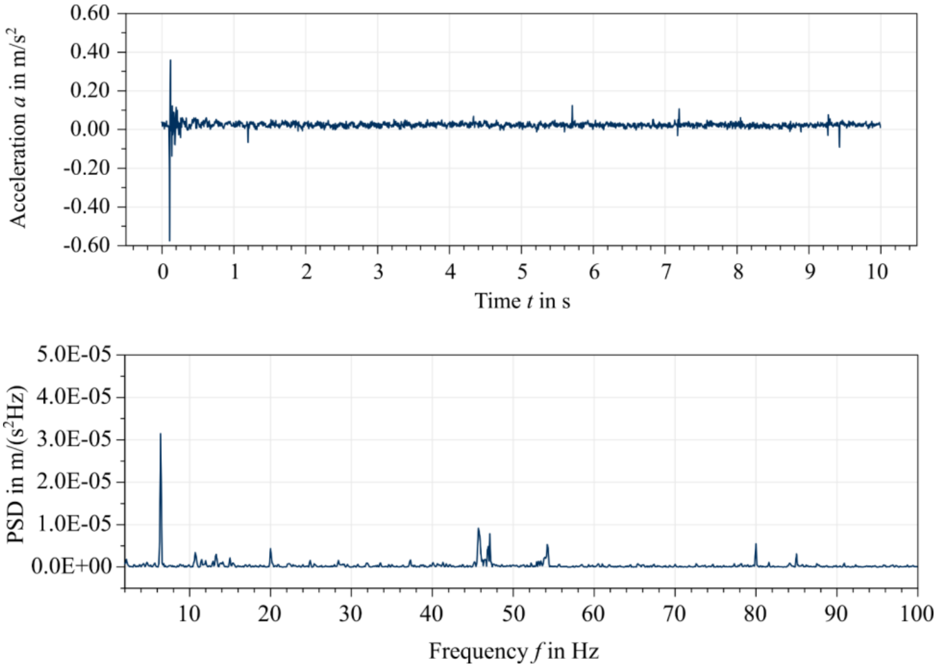

Fig. 6. Typical recorded monitoring data (acceleration measurements) due to ambient excitation: Acceleration measurements (top); power spectral density function according to the Welch method and flat-top windowing (bottom) (Graphic: Jan-Hauke Bartels).

The results of the system identification are shown in Fig. 7, where the evolution of the eigenfrequency is plotted over time. It can be clearly seen that the eigenfrequency of the structure changes over time, especially due to changes in the static system. Eigenfrequencies between 4.5 Hz and 7 Hz were measured over the entire period. A comparison with the temperature shows fluctuations between 5 °C and 20 °C during the considered monitoring period. At the beginning of the measurements (t = -10 d), the bridge was under construction and the PEs were placed at their final positions. Temporary scaffolding near axis 20 reduces spans from 15 m to 12.5 m. In this state, the PEs of span 1 and span 2 are decoupled from each other and not yet monolithically connected to the substructure, allowing the PEs of both spans to move separately. It is observed that despite equal spans the eigenfrequency in span 1 is lower than that in span 2, which is due to the lower concrete compressive strength and Young's modulus of span 1 (C25/30) compared to span 2 (C50/60). On day 0, the in-situ concrete layer was cast to form the overall cross-section of the slab and the monolithic connections between the abutment in axis 10, the piers in axis 20 and the superstructure. After concreting, the eigenfrequency drops significantly. This can be explained by the additional mass on the system without an increase in stiffness. After the scaffolding has been lowered on day 22, a decrease in the eigenfrequency can be observed. As the concrete continues to hydrate, the two separate PEs became a two-span beam with a rigid frame corner in axis 10, resulting in an increase in eigenfrequency until day 40. During the same period, the temperature increases, which should theoretically reduce the eigenfrequency. With increasing measurement duration, the effects of temperature increase (compared to concrete hardening) become predominant and the eigenfrequency between t = 40 d and t = 72 d decreases by about 20 %. It can be noted that the eigenfrequencies of the two spans approach each other over time, which shows that the originally individual spans are merging into one system due to progressive concrete hardening. A comparison measurement by the Hamburg University of Technology (TUHH) on day 72 with a different piezoelectric accelerometer shows that small differences in the determination of the eigenfrequencies can occur due to the use of different sensors types, but in principle the determined eigenfrequencies can be verified. Since the environmental excitation causes relatively weak vibrations on the superstructure, the dynamic behavior of the structure is investigated at the end of the temporary measurements on day 72 with higher amplitudes by

determining the eigenfrequency with a head point excitation as a result of an impulse hammer impact. The hammer impact measurement confirms the ambient excitation measurements.

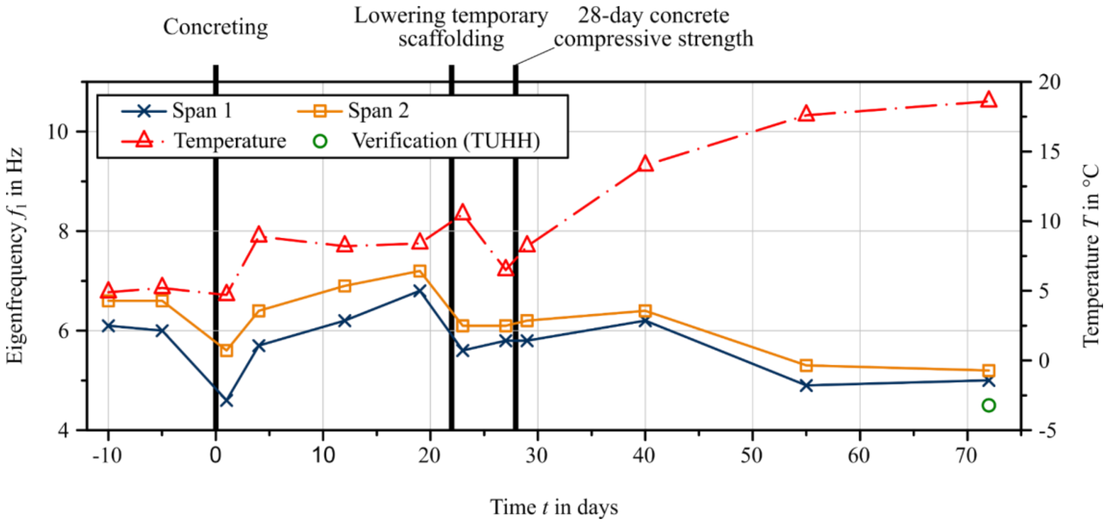

Fig. 7. Evolution of the eigenfrequency over time (y_1-axis) and temperature over time (y_2-axis) (Graphic: Jan-Hauke Bartels).

5 Conclusions and future work

The IDA-KI research project presents a crucial step towards automated evaluation and assessment of monitoring data for civil infrastructure. The openLAB, a research bridge constructed in Bautzen, Germany, serves as an integral case study. The bridge is equipped with a comprehensive SHM system, and offers a unique opportunity to validate structural evaluation methods using real-world data that encompass both structural damage and sensor faults. During the construction process of the openLAB bridge, the evolution of the eigenfrequency has been recorded and can be used in the future as a reference, indicating possible structural damage. The monitoring data is made available at regular intervals via a data repository. Information can be obtained from the project website [7], which also provides further information on the openLAB bridge (e.g., material parameters and construction drawings) and the installed SHM system. In the next step, the bridge will be severely damaged in various loading tests after the one-year reference phase. The monitoring data collected during the loading tests will be stored in a database, which contains specific signal characteristics of structural damage, and will be used to test and further develop the evaluation methods presented in Section 3. In the future, the evaluation methods should enable reliable anomaly detection and differentiation between structural changes and faults in the SHM system.

Acknowledgements

This paper presents part of the results and findings of the IDA-KI research project. The authors would like to gratefully acknowledge the support offered by the Federal Ministry for Digital and Transport (BMDV), Germany, within the mFUND program under grant 19FS2013A-D. The authors would also like to thank the project partners Hentschke Bau GmbH and MKP GmbH for their valuable cooperation. Any opinions, findings, conclusions, or recommendations expressed in this paper are those of the authors and do not necessarily reflect the views of the BMDV.

References

1. Smarsly, K., and Law, K. H., “Decentralized fault detection and isolation in wireless structural health monitoring systems using analytical redundancy,” Advances in Engineering Software, V. 73, 2014, pp. 1–10.

2. Herbers, M., Bartels, J.-H., Richter, B., Collin, F., Ulbrich, L., Al-Zuriqat, T., Chillón Geck, C., Naraniecki, H., Hahn, O., Jesse, F., Smarsly, K., and Marx, S., "openLAB – Eine Forschungsbrücke zur Entwicklung eines digitalen Brückenzwillings," Beton- und Stahlbetonbau, V. 119, No. 3, 2024, pp. 169–180.
3. Collin, F., Ulbrich, L., and Jesse, F., "Konzept eines Digitalen Zwillings für Brückenbauwerke in der Betriebsphase," Bautechnik, V. 101, No. 3, 2024, pp. 199–205.
4. Richter, B., Messerer, D., Herbers, M., Speck, K., Laukner, J., Gläser, C., Jesse, F., and Marx, S., "Monitoring of a prestressed bridge girder with integrated distributed fiber optic sensors" Proceedings of the 7th International Conference on Smart Monitoring, Assessment and Rehabilitation of Civil Structures (SMAR), NN, ed., Springer, 2024.
5. Brühwiler, E., "UHPFRC technology to enhance the performance of existing concrete bridges," Structure and Infrastructure Engineering, V. 16, No. 1, 2020, pp. 94–105.
6. Chalouhi, E. K., Gonzalez, I., Gentile, C., and Karoumi, R., "Vibration-Based SHM of Railway Bridges Using Machine Learning: The Influence of Temperature on the Health Prediction," Experimental Vibration Analysis for Civil Structures: Testing, Sensing, Monitoring, and Control, 1st ed. 2018, J. P. Conte, R. Astroza, G. Benzoni, G. Feltrin, K. J. Loh, and B. Moaveni, eds., Springer International Publishing, Cham, 2018, pp. 200–211.
7. openLAB website, https://tu-dresden.de/bu/bauingenieurwesen/imb/forschung/ida-ki-infrastrukturdatenauswertung-mit-kuenstlicher-intelligenz?set_language=en, last accessed 2024/04/19.
8. Herbers, M., Richter, B., Gebauer, D., Classen, M., and Marx, S., "Crack Monitoring on Concrete Structures – Comparison of Various Distributed Fiber Optic Sensors with Digital Image Correlation Method," Structural Concrete, V. 24, No. 5, 2023, pp. 6123–40.
9. Zhang, S., Liu, H., Coulibaly, A. A. S., and DeJong, M., "Fiber optic sensing of concrete cracking and rebar deformation using several types of cable," Struct Control Health Monit, V. 28, No. 2, 2020.
10. Liu, Y., Li, X., Li, H., and Fan, X., "Global Temperature Sensing for an Operating Power Transformer Based on Raman Scattering," Sensors, V. 20, No. 17, 2020, p. 4903.
11. Piątek, B., Howiacki, T., Kulpa, M., Siwowski, T., Sieńko, R., and Bednarski, Ł., "Strain, crack, stress and shape diagnostics of new and existing post-tensioned structures through distributed fibre optic sensors," Measurement, V. 221, 2023.
12. Zhang, Z., Mehmood, A., Shu, L., Huo, Z., Zhang, Y., and Mukherjee, M., "A Survey on Fault Diagnosis in Wireless Sensor Networks," IEEE Access, V. 6, 2018, pp. 11349–11364.
13. Frank, P. M., "Fault diagnosis in dynamic systems using analytical and knowledge-based redundancy," Automatica, V. 26, No. 3, 1990, pp. 459–474.
14. Samy, I., Postlethwaite, I., and Gu, D.-W., "Survey and application of sensor fault detection and isolation schemes," Control Engineering Practice, V. 19, No. 7, 2011, pp. 658–674.
15. Patton, R. J., "Fault detection and diagnosis in aerospace systems using analytical redundancy," Comput. Control Eng. J., V. 2, No. 3, 1991, pp. 127–136.
16. Al-Zuriqat, T., Chillón Geck, C., Dragos, K., and Smarsly, K., "Adaptive Fault Diagnosis for Simultaneous Sensor Faults in Structural Health Monitoring Systems," Infrastructures, V. 8, No. 3, 2023, p. 39.
17. Al-Zuriqat, T., Al-Nasser, H., Dragos, K., Geck, C., and Smarsly, K., "Identification of composite sensor faults in structural health monitoring systems using long short-term memory networks," Proceedings of the 2024 European Conference on Computing in Construction (EC3), 2024.
18. Isermann, R., and Ballé, P., "Trends in the application of model-based fault detection and diagnosis of technical processes," Control Engineering Practice, V. 5, No. 5, 1997, pp. 709–719.
19. Al-Zuriqat, T., Peralta Abadia, P., Chillón Geck, C., Dragos, K., and Smarsly, K., "Implementation and validation of a low-cost IoT-enabled shake table system," Proceedings of the 14th International Workshop on Structural Health Monitoring (IWSHM), S. Farhangdoust, A. Guemes, and F.-K. Chang, eds., DEStech Publications, Inc, 2023, pp. 1063–1070.
20. Al-Zuriqat, T., Chillón Geck, C., Dragos, K., and Smarsly, K., "Diagnosis of simultaneous sensor faults in structural health monitoring systems," Proceedings of the 2023 European Conference on Computing in Construction and the 40th International CIB W78 Conference, European Council for Computing in Construction, 2023.
21. Richter, B., Herbers, M., and Marx, S., "Crack monitoring on concrete structures with distributed fiber optic sensors – Toward automated data evaluation and assessment," Structural Concrete, V. 25, No. 2, 2023, pp. 1465-1480.
22. Janiak, T., Becks, H., Camps, B., Classen, M., and Hegger, J., "Evaluation of distributed fibre optic sensors in structural concrete," Mater Struct, V. 56, No. 9, 2023.
23. Richter, B., "fosanalyis: A framework to evaluate distributed fiber optic sensor data," 2023.
24. Bado, M. F., Casas, J. R., and Gómez, J., "Post-processing algorithms for distributed optical fiber sensing in structural health monitoring applications," Structural Health Monitoring, V. 20, No. 2, 2021, pp. 661–680.
25. Fischer, O., Thoma, S., and Crepaz, S., "Distributed fiber optic sensing for crack detection in concrete structures," Civil Engineering Design, V. 1, 3-4, 2019, pp. 97–105.
26. Berrocal, C. G., Fernandez, I., and Rempling, R., "Crack monitoring in reinforced concrete beams by distributed optical fiber sensors," Structure and Infrastructure Engineering, V. 17, No. 1, 2021, pp. 124–139.
27. Bartels, J.-H., Xu, R., Kang, C., Herrmann, R., and Marx, S., "Experimental Investigation on the Transfer Behavior and Environmental Influences of Low-Noise Integrated Electronic Piezoelectric Acceleration Sensors," Metrology, V. 4, No. 1, 2024, pp. 46–65.
